# Modeling treatment-dependent glioma growth including a dormant tumor cell subpopulation

**DOI:** 10.1186/s12885-018-4281-1

**Published:** 2018-04-03

**Authors:** Marvin A. Böttcher, Janka Held-Feindt, Michael Synowitz, Ralph Lucius, Arne Traulsen, Kirsten Hattermann

**Affiliations:** 1 Department Evolutionary Theory, Max Planck Institute for Evolutionary Biology, 24306 Plön, Germany; 2 Department of Neurosurgery, University Medical Center Schleswig-Holstein UKSH, Campus Kiel, 24105 Kiel, Germany; 3 Department of Anatomy, University of Kiel, 24098 Kiel, Germany

**Keywords:** Evolutionary game theory, Glioma, Dormancy

## Abstract

**Background:**

Tumors comprise a variety of specialized cell phenotypes adapted to different ecological niches that massively influence the tumor growth and its response to treatment.

**Methods:**

In the background of *glioblastoma multiforme*, a highly malignant brain tumor, we consider a rapid *proliferating* phenotype that appears susceptible to treatment, and a *dormant* phenotype which lacks this pronounced proliferative ability and is not affected by standard therapeutic strategies. To gain insight in the dynamically changing proportions of different tumor cell phenotypes under different treatment conditions, we develop a mathematical model and underline our assumptions with experimental data.

**Results:**

We show that both cell phenotypes contribute to the distinct composition of the tumor, especially in cycling low and high dose treatment, and therefore may influence the tumor growth in a phenotype specific way.

**Conclusion:**

Our model of the dynamic proportions of dormant and rapidly growing glioblastoma cells in different therapy settings suggests that phenotypically different cells should be considered to plan dose and duration of treatment schedules.

## Background

Gliomas are the most common type of primary brain tumors including their highly malignant form, the *glioblastoma multiforme* (GBM), which accounts for about 15% of all brain tumors [1]. Despite current standard treatment of GBM by surgical resection and adjuvant radio- and chemotherapy, the median survival time for GBM patients is still poor, approximating 12–15 months [2], mostly due to unsatisfactory response of the tumor to treatment strategies. Additionally, combined aggressive radio−/chemotherapy is causing severe side effects frequently necessitating interruptions of the therapy due to e.g. blood toxicity [3]. GBMs and also many other tumors are heterogeneous tumors, being composed of cells with different, partly specialized phenotypes [4]. Besides e.g. rapidly proliferating tumors cells, invading immune cells, endothelial cells and (tumor) stem cells, also a subpopulation of so called *dormant* tumor cells exists in the heterogeneous tumor mass. These cells enter a quiescent state driven by cell-intrinsic or extrinsic factors, including permanent competition for nutrients, oxygen, and space (“cellular dormancy”) [5–8]. In several tumors and metastases, dormant cells have been shown to be not proliferative or only very slowly cycling [9–12]. Linking dormancy and effects of chemotherapy, studies on glioma cells showed that cells underwent a prolonged cell cycle arrest upon treatment with temozolomide (TMZ), the most common chemotherapeutic in GBM therapy [13].

Evolutionary forces, such as competition and selection, shape the growth of the tumor and therefore the progression of the cancer. These forces create different ecological niches within the tumor encouraging the adaption of specialized tumor cell phenotypes. Accordingly, the proportional balance between different tumor cellular phenotypes can drastically change with treatment conditions. Indeed, compared to rapidly proliferating tumor cells, especially dormant cells exhibit a much higher robustness against chemotherapeutic drugs [5]. This dormant state seems to be reversible [13], so that the conversion to dormancy and the exit from dormancy may be a mechanism that facilitates tumor survival and progression even upon adverse or changing conditions. Hence, a better understanding of the proportional dynamics of different cell phenotypes within gliomas under chemotherapeutic treatment may improve further therapeutic approaches.

Mathematical models are beneficial resources to gain insight into key mechanisms of cancer development, growth, and evolution and to help identifying potential therapeutic targets [14]. Among these approaches, evolutionary game theory [15, 16] models the interactions between different individuals as a game between agents playing different strategies and relates the payoff from this game to the reproductive fitness of the corresponding agent [17–21].

Here, we use evolutionary game theory to model the proportions of two different phenotypes of GBM cells in a variety of different treatment conditions, see Basanta and Deutsch [18] for a related approach in GBM. Defining the fitness of the different cell types as growth rate in comparison to cells of the respective other phenotype, we focus especially on the balance between the rapidly proliferating and the cellular dormant phenotype and describe the corresponding payoffs in a payoff matrix which also includes the effect of treatment. Then, we use a special form of the replicator-mutator equation [22, 23], which takes into account that conversion from dormant to rapidly proliferating phenotype and *vice versa* is possible. To strengthen our theoretical assumptions, we analyzed cell numbers and the cellular expression of a dormancy marker under different chemotherapy dosages and the phenotypic conversion modalities in cultured GBM cells in vitro. Taken together, the aim of our study was to develop a simple theoretical model which describes the dynamically changing proportions of two different GBM cell phenotypes, rapidly proliferating and dormant cells, under different treatment conditions. Showing this, we suggest that different properties of cell phenotypes should be taken into account for the development of more efficient, less toxic treatment schedules in order to improve patient’s prognosis and quality of life.

## Methods

### Theoretical model

We analyze the proportions of two different GBM cell phenotypes, dormant (D, please refer to Table 1 for symbols used in the equations) and rapidly proliferating (P) cells, in a mathematical model including the influence of different treatment conditions. In the following, we characterize the cells in terms of their fitness, which we define as the growth rate in comparison to cells of the other phenotype. Dormant cells always have a very low or even zero growth rate *ε*, which we assume to be independent of the exact composition of the population and the treatment condition. Rapidly proliferating P cells, on the other hand, have a very large fitness advantage compared to dormant cells, which means they proliferate much faster, but they also compete with each other for space and resources. Facing another P cell, a focal P cell has an intermediate fitness, which we assume to be still much larger than the growth rate of D cells *ε*. Their fitness therefore depends on the relative fraction of D vs. P cells. Due to the very slow growth of D cells, P cells will represent the vast majority of glioblastoma cells in the absence of treatment.

**Table 1:** Overview of all used symbols in the model

*n*_*X*_	number of cells of type *X*
*x*_*X*_	ratio of cells of type *X* in population
*D*, *P*	index for dormant or rapidly proliferating cell type, respectively
*ε*	Fitness of dormant (*D*) cells
*λ*	treatment cost on normally growing cells
*σ*	probability for spontaneous conversion between types
$\overline{f}$	total average fitness of all cell types in the population

Under treatment conditions, however, the population composition changes. Even though D cells still have the same very low (or zero) growth rate *ε*, P cells experience a fitness cost *λ* due to treatment. The reduction of the fitness due to treatment only applies to P cells, because cytotoxic drugs mostly affect rapidly dividing cells. The fitness cost parameter *λ* can be adjusted to account for the strength of the applied treatment. In principle we can continuously vary this parameter. However, for simplicity we focus on two different treatment strategies: In high dosage (HD) chemotherapy the treatment strength parameter *λ* is large compared to the growth rate of the P type. Since high dosage chemotherapy has strong side effects for the whole organism (for GBM: [3]), in reality this treatment strategy cannot be maintained for extended time periods. Therefore, strong treatment needs to be applied in turns with weaker or no treatment. For low dosage (LD) chemotherapy, *λ* means only a small reduction of the growth rate of the P cells. As the side-effect stress to the organism should also be lower, this treatment regime could be applied for longer time spans.

Dormant (D) and rapidly proliferating (P) phenotypes in glioblastoma and their aforementioned interactions can be described by the following payoff matrix [18]:

$$ {\displaystyle \begin{array}{c}\\ {}\mathrm{D}\\ {}\mathrm{P}\end{array}}{\displaystyle \begin{array}{c}\mathrm{D}\kern1.50em \mathrm{P}\\ {}\left(\begin{array}{cc}\varepsilon & \varepsilon \\ {}1-\varepsilon -\lambda & \frac{1}{2}-\lambda \end{array}\right)\end{array}} $$

This matrix gives the fitness for each type if confronted with any of the two other types. Here, we find for example that the fitness of a focal P cell interacting with a D cell is 1 − *ε* − *λ*, which includes both the small or zero growth rate of D cells *ε* and the fitness cost for P cells under treatment *λ*.

As the phenomenon of dormancy is presumably a reversible process that also occurs without any treatment, we assume that conversion between both phenotypes is possible with a small rate *σ*. Thus, P cells may enter a dormant phenotype, and D cells may exit from their quiescent state, converting into a P phenotype at any time point.

In the following, we include these fitness effects and phenotypic conversion into a set of ordinary differential equations. In general, the growth of a whole cell population can be explained in terms of a differential equation that describes the change in the number of individuals over time

$$ \frac{dn}{dt}=r\left(n,t\right)n. $$

Here *n* is the number of individuals, *t* is the time and *r*(*n*, *t*) is the growth rate, which can itself depend on the number of cells and the time.

At first, we focus on the number of D cells, *n*_*D*_, in the population over time, which have a very small but constant growth rate *ε*

$$ \frac{d{n}_D}{dt}=\varepsilon\ {n}_D. $$

For P cells on the other hand, the growth rate of *n*_*P,*_ given by the average fitness from the payoff matrix (weighted to the cell fractions), changes with the composition of the population

$$ \frac{d{n}_{\mathrm{P}}}{dt}={n}_{\mathrm{P}}\left(\left(1-\varepsilon -\lambda \right)\frac{n_D}{n_D+{n}_{\mathrm{P}}}+\left(\frac{1}{2}-\lambda \right)\frac{n_{\mathrm{P}}}{n_D+{n}_{\mathrm{P}}}\right). $$

Since the system under consideration is constrained, both in terms of nutrients and space, in reality the cell population only grows exponentially as indicated by the growth equations in the very beginning of the process where the constraints regarding space or nutrients are negligible. However, we are mainly interested in the fraction of D cells ${x}_D=\frac{n_D}{n_D+{n}_{\mathrm{P}}}$ in the population and vice versa the fraction of P cells ${x}_{\mathrm{P}}=1-{x}_D=\frac{n_{\mathrm{P}}}{n_D+{n}_{\mathrm{P}}}$. To obtain the change in fractions for both types, we subtract the average growth rate $\overline{f}$ of the population from both individual growth rates,

$$ \overline{f}\kern0.5em =\varepsilon {x}_D+\left[\left(1-\varepsilon -\lambda \right){x}_D+\left(\frac{1}{2}-\lambda \right){x}_{\mathrm{P}}\right]{x}_{\mathrm{P}} $$

From this we obtain two differential equations for the fractions of D and P cells,

$$ {\displaystyle \begin{array}{cl}{\dot{x}}_D& ={x}_D\left(\varepsilon -\overline{f}\right)\\ {}{\dot{x}}_{\mathrm{P}}& ={x}_{\mathrm{P}}\left(\left[\left(1-\varepsilon -\lambda \right){x}_D+\left(\frac{1}{2}-\lambda \right){x}_{\mathrm{P}}\right]-\overline{f}\right)\end{array}} $$

Next, we include the spontaneous conversion between phenotypes with a constant rate *σ*, which is independent of the cellular growth. This leads to an additional term to the differential equation of both phenotypes

1 $$ {\displaystyle \begin{array}{cl}{\dot{x}}_D& =\left[\varepsilon -\overline{f}\right]{x}_D+\sigma \left({x}_{\mathrm{P}}-{x}_D\right)\\ {}{\dot{x}}_{\mathrm{P}}& =\left[\left(1-\varepsilon -\lambda \right){x}_D+\left(\frac{1}{2}-\lambda \right){x}_{\mathrm{P}}-\overline{f}\right]{x}_{\mathrm{P}}+\sigma \left({x}_D-{x}_P\right)\end{array}}. $$

These equations have the important difference to the usual replicator-mutator equation [15] that phenotype conversion is a spontaneous process with a constant rate and is independent of the growth in the population. This allows conversion from D to P even if D cells do not grow at all.

Using these equations, we model different therapy schedules combining different treatment strengths in different cycling time plans. Since the equations are nonlinear, we use numerical integration with *Odeint* of the Python library Scipy^1^ to examine the temporal dynamics of the system under different treatment regimes. Additionally we analytically determine the fixed points of the system and their stability.

### Experimental model

#### Cell culture and cell number determination

The GBM cell line LN229 was purchased from ATCC/LGC Standards (Middlesex, UK, ATCC-CRL 2611) and cultured in Dulbecco’s modified eagle medium (DMEM) plus 10% fetal calf serum (FCS, PAN Biotech, Aidenbach, Germany). *Mycoplasma* contaminations were routinely excluded by bisbenzimide staining. The GBM cell line identity was proven routinely by STR (Short Tandem Repeat) profiling at the Department of Forensic Medicine (Kiel, Germany) using the Powerplex HS Genotyping Kit (Promega, Madison, WC). Briefly, DNA was amplified with a STR multiplex PCR, electrophoretic separation was performed with the 3500 Genetic Analyser (Thermo Fisher Scientific, Waltham, MA, USA), and evaluated using the Software GeneMapper ID-X (Thermo Fisher Scientific). For determination of cell numbers after low and high dose chemotherapy treatment, 25,000 cells/well were seeded in 6 well plates (Greiner Bio-one, Frickenhausen, Germany). Cells were grown for 24 h, then washed with phosphate buffered saline (PBS), supplemented with fresh DMEM + 10% FCS and temozolomide concentrations (Sigma-Aldrich, St. Louis, MO, USA; dissolved in dimethyl sulfoxide DMSO) as indicated in Fig. 2a (5, 50 or 100 μg/ml for 10 days). Temozolomide (TMZ) is a DNA alkylating drug causing apoptotic cell death and the most commonly used chemotherapeutic in GBM therapy. Control cells were supplemented with 0.5% DMSO, which corresponds to the solvent concentrations of each TMZ stimulated sample. Cells were stimulated for 10 days with TMZ, while media were changed every 2–3 days. After 10 days, cells were detached by trypsination and total cell numbers per well counted using trypan blue exclusion and a Neubauer chamber (Brand, Wertheim, Germany). DMSO stimulated control cells were already detached after 6 days of stimulation, split 1:10 and seeded again to exclude limitations of growth due to space and nutrient limitations. This splitting factor (1:10) was considered when relative cell numbers of TMZ treated samples in comparison to DMSO controls were determined for *n* = 5–6 independent experiments.

#### Immunocytochemistry

For immunocytochemistry, 50,000 cells were seeded onto poly-D-lysine coated glass cover slips, grown for 24 h and supplemented with indicated TMZ or DMSO concentrations as described above. From day 6, growth media were additionally supplemented with 10 μM 5-bromo-2′-deoxyuridine (BrdU, Sigma-Aldrich, St. Louis, MO) to allow for incorporation in the DNA in the S phase of the cell cycle. After 10 days, cover slips were fixed with an ice-cold mixture of methanol and acetone (1:1) for 10 min, rinsed with 0.1% Tween / PBS (3 × 5 min), incubated with 1 M HCl for 30 min, neutralized with 0.1 M sodium borate buffer (pH 8.5), and rinsed again with 0.1% Tween/PBS. Afterwards, cells were blocked for unspecific bindings with 0.5% bovine serum albumin (BSA) / 0.5% glycine in PBS (1 h) and incubated over night with the primary antibody against H2BK (1:300, Biorbyt, Cambridge, UK), a marker of glioma dormancy [24, 25] and the primary antibody against BrdU (1:200, Abcam, Cambridge, UK). Then cover slips were incubated with the secondary antibodies (donkey anti-rabbit IgG, labelled with Alexa Fluor 488, and donkey anti-sheep labelled with Alexa Fluor 555, both Invitrogen, Carlsbad, CA, USA) for 1 h at 37°, and 4′, 6-diamidino-2-phenylindole (DAPI; Sigma Aldrich, St. Louis, MO, USA; 1 mg/ml, 1:30,000, 30 min at room temperature) to stain nuclei. Cover slips were embedded using Immu-Mount (Thermo Fisher Scientific, Rockford, IL, USA), and analysed with equal exposure times using an Axiovert microscope and digital camera (Zeiss, Jena, Germany). H2BK-immunopositive, BrdU-positive and double positive cells were counted and normalized to total cell numbers in 6 (DMSO controls) to 10 (TMZ samples) fields of view for *n* = 4 independent experiments.

#### DiO retention and cell countings on phenotype conversion

To monitor the conversion to and from dormancy we used the green fluorescent vital dye DiO (Invitrogen), as rapidly proliferating cells lose the dye due to repeated divisions, while resting, dormant (or very slowly cycling) cells retain the dye and can be detected by fluorescence microscopy. Investigating the conversion to dormancy, 150,000 LN229 cells were seeded into 6-well-plates, stained with Vybrant® DiO Cell-Labeling Solution (Thermo Fisher Scientific, Waltham, MA, USA) following the manufacturer’s instructions and stimulated with 100 μg/ml TMZ (or equal volume of the solvent DMSO) for 10–12 days. Cells were photographed combining transmitted-light microscopy and fluorescence microscopy with equal exposure times for TMZ and control treated cells, and green fluorescent cell portions were determined in comparison to total cell counts. To determine the influence of different cell densities on the incidence of conversion, 50,000 and 150,000 cells were seeded, respectively, into 6-well-plates and treated with 100 μg/ml TMZ (or equal volumes of the solvent DMSO) for 10 days. As the DMSO control treated cells rapidly proliferate, cells were detached at day 6 (50,000) or day 3 and 6 (150,000), cell numbers counted using a Neubauer chamber to determine the growth rate over this time period, and seeded again at initial density, to allow for cell growth without limitation of space and nutrients. After 10 days, TMZ and control treated cells were detached and counted. To extrapolate the total cell numbers of control cells, growth rates determined at day 3, 6 and 10 were used, and TMZ surviving cells were calculated as percentage of extrapolated total cells.

#### Statistical analysis

Statistical analysis and graphical presentation of experimental data were performed with Graph Pad Prism using a two-tailed t-test (*** *p* < 0.001).

## Results

### Modelling the dynamics of cell frequencies

The temporal dynamics of the proportion of D against P cells in GBM strongly depends on the treatment conditions. Therefore, we first analyze the fixed points of the dynamical system and how they change for different treatment strengths *λ*, without considering possible conversions of phenotypes. The fixed points mark a stable equilibrium between the portions of P and D cells under certain, predefined conditions and are found by setting Eq. 1 to zero. Of particular interest are *stable* fixed points, as the system returns into this state again after a small perturbation [26].

**Fig. 1:**
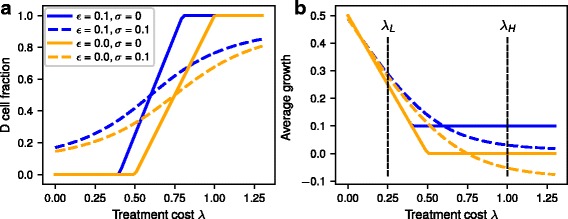
**a** Equilibrium fraction of dormant cells depending on treatment cost *λ*. **b** Average growth rate at the fixed point depending on treatment cost *λ*. Blue lines indicate a growth rate of D cells of *ε* = 0 and orange lines a growth rate *ε* = 0.1. Solid lines are for the absence of phenotype conversion (*σ* = 0) and dashed lines with phenotype conversion (*σ* = 0.1)

For our system, there is only one stable fixed point for each treatment condition (Fig. 1a). If we consider the case of no phenotype conversion, *σ* = 0, we can give the exact position of this point for each treatment condition *λ*. The fraction of *D* cells at the fixed point is then given by

$$ {x}_D\kern0.5em =\Big\{{\displaystyle \begin{array}{ll}0& \frac{2\lambda }{1-2\varepsilon}\le 1\\ {}\frac{2\lambda }{1-2\varepsilon }-1& 1\le \frac{2\lambda }{1-2\varepsilon}\le 2\\ {}1& 2\le \frac{2\lambda }{1-2\varepsilon}\end{array}}\operatorname{} $$

For small treatment strengths *λ* the fraction of D cells in the population at the stable fixed point is zero, but after reaching a threshold, the fraction of dormant cells increases linearly with *λ* until the whole population consists of dormant cells at very high treatment strengths.

With conversion between the two phenotypes (*σ* > 0), the analytical calculation for the stable fixed points is more difficult and the result is not instructive. By contrast to the previous results without phenotype conversion, there is always a small proportion of dormant cells in the population, even at very low treatment strengths. The proportion of dormant cells at the fixed points increases immediately with increasing treatment strength until it approaches a maximum at high treatment strengths. For both D cell growth rates *ε* (*ε* = 0, orange lines; and *ε* = 0.1, blue lines) the population composition is very similar or even the same without phenotype conversion at very small or large treatment strengths *λ*. In contrast, the largest effect of *ε* on the population composition is at intermediate values of treatment strength.

The average fitness $\overline{f}$ for the whole tumor cell population including P and D cells decreases linearly from the maximum at treatment strength *λ* = 0 until it reaches the minimum of $\overline{f}=\varepsilon$ at the point where the fraction of dormant cells in the population starts to increase (Fig. 1b). Interestingly, with spontaneous conversion *σ* > 0, the average fitness at the fixed point can become smaller than *ε* and even negative for high treatment strengths, potentially leading to a shrinking tumor. This is caused by conversion of D cells into P cells which are then susceptible to treatment.

### Comparison to experimental data

**Fig. 2:**
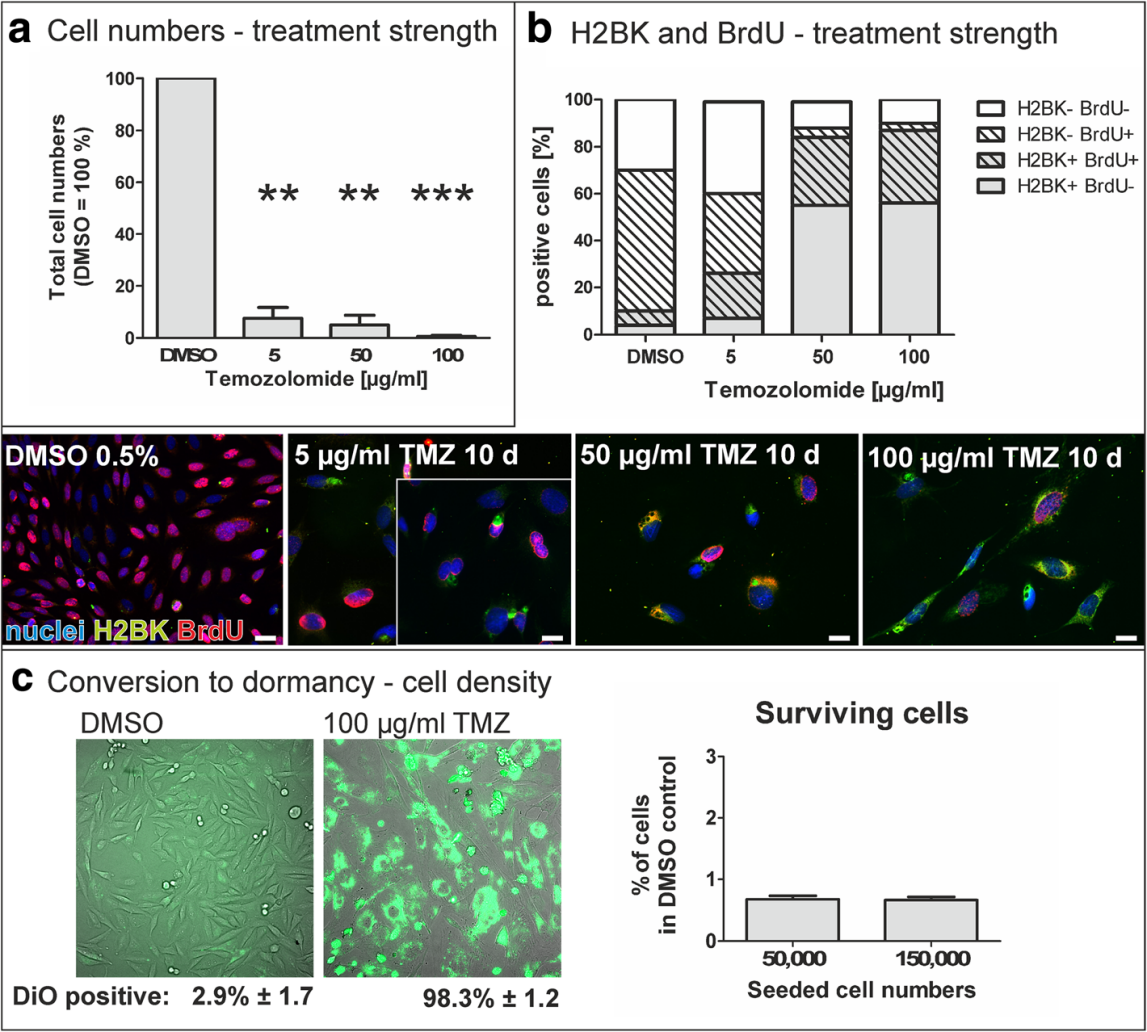
**a** Decrease of total cell numbers upon different temozolomide (TMZ) treatment strength. LN229 glioma cells were treated with different TMZ concentrations for 10 days, control cells were treated with the solvent DMSO (0.5%). Total cell numbers strongly decreased in a TMZ concentration dependent manner. Given are mean values of cell counting ± SD from *n* = 3 independent experiments. **b** Increase of the H2BK positive dormant cell portion upon different TMZ treatment strength, and incorporation of BrdU. The fraction of dormant cells as determined by immunoreactivity for the glioma dormancy marker H2BK and counting of the positively stained cells was remarkably increased in a concentration dependent manner (grey portions of the graph). The fraction of cells with BrdU incorporation in turn decreased (hatched portions of the columns), but, remarkably, in higher TMZ concentrations, H2BK and BrdU double positive cells were frequently observed (hatched, grey portions of the columns). Microscopic pictures exemplarily show cells expressing the dormancy marker H2BK (green) and the incorporation of BrdU (red) upon stimulation with different concentrations of TMZ for 10 days. The pictures are representative examples from 6 to 10 fields of view that were analyzed for *n* = 4 independent experiments and summarized in the graphs in the upper part; the bars indicate 20 μm. **c** Influence of cell density on portions of dormant cells. Left: When stained with the vital dye DiO and treated with 100 μg/ml TMZ for 10–12 days, nearly all cells (about 98%) cease from dividing which is indicated by the retention of the green fluorescent dye. Meanwhile, in control treated cells (DMSO), nearly all cells lose the green fluorescent dye due to repeated cell divisions. Right: Graphs show surviving dormant cells after 10 days of TMZ treatment (100 μg/ml) in dependence of the initially seeded cell numbers. Portions of surviving cells are very low in comparison to total (extrapolated) cell numbers in DMSO control cultures, and do not depend on initially seeded cell numbers. Given are mean values of cell counting ± SD from *n* = 6 independent experiments

To test our mathematical model of phenotype composition upon treatment, we used LN229 cells as an experimental in vitro model. We treated these cells for 10 days with temozolomide (TMZ), the most commonly used cytotoxic drug in glioma therapy. In a first step, we focused on different treatment strength and analysed the portions of surviving cells in comparison to control cultures and the percentage of cells expressing H2BK (histone cluster 1), a marker of glioma dormancy [24, 25], alongside with incorporation of BrdU in the late treatment phase (day 6–10). In general, after 10 days of treatment, samples stimulated with 5, 50 and 100 μg/ml TMZ had significantly less total cell numbers than control treated cells (Fig. 2a). By immunocytochemistry of H2BK, we could detect and quantify the fraction of dormant cells within the cultures, and by adding BrdU to the cells from day 6 of treatment and immunocytochemical staining of BrdU, we could in parallel mark cells that incorporate BrdU in the DNA (examples of microscopic pictures in Fig. 2b). While DMSO-treated control cells showed a low fraction of H2BK-positive cells (mean: 9.7% ± 3.5), TMZ treatment yielded increased numbers of dormant cells reaching a plateau at high concentrations (5 μg/ml: mean 26.8% ± 9.0, 50 μg/ml: mean: 82.8% ± 5.3, 100 μg/ml: 87.7% ± 8.0, compare Fig. 2b, grey graph portions). In parallel, we investigated the incorporation of BrdU in the DNA and determined a high portion (66.0% ± 7.8) of BrdU positive cells in the control cultures and lower portions upon TMZ treatment (5 μg/ml: 53.6% ± 14.5; 50 μg/ml: 33.4% ± 5.3; 100 μg/ml: 33.7% ± 10.1, compare Fig. 2b, hatched graph portions). Interestingly, BrdU incorporation also took place in TMZ treated cultures, so that staining for the dormancy marker H2BK and for BrdU could be observed in the very same cells (compare examples of microscopic photographs in Fig. 2b) indicating that cell cycle arrest may occur after the S phase of the cell cycle. Together with our experiments described in the following section and Fig. 2c, showing that dormant cells hardly divide within our experimental time frame, these observations suggest that dormant glioma cells are not or only very slowly cycling. Furthermore, taking into account that we use a clonal cell line, the occurrence of dormant cells needs to be a phenotypic adaption to the environmental conditions as all cells are genetically homogenous (as proven by routinely STR profiling, compare Materials and Methods section).

To investigate if the conversion to a dormant phenotype depended on the cell density, initially, we determined in a DiO retention assay that nearly all cells (98.3% ± 1.2) retain the green fluorescent dye when treated with 100 μg/ml (“high dose”) TMZ for 10–12 days, while in control cultures (treated with equal volumes of the solvent DMSO) only 2.9% ± 1.7 retained the dye (Fig. 2c, left part). The vital dye is included in every cell at the moment of staining, and is transferred to every daughter cell upon cell division. However, this means the staining is diminishing after several divisions of rapidly proliferating cells, but retained in non-proliferative or very slowly cycling dormant cells. Thus, assuming that nearly all cells that survive treatment with 100 μg/ml TMZ are dormant in our particular setting, we determined the relative incidence of phenotype conversion and the influence of the cell density on this conversion factor by determination of TMZ surviving cells in relation to (extrapolated) total cell numbers of control (DMSO treated) cultures. In our experimental setting, a portion of 0.68% ± 0.13 cells of initially seeded 50,000 LN229 glioblastoma cells survived this high dose treatment, while in cultures of initially seeded 150,000 cells, the portion of surviving cells was nearly similar (0.66% ± 0.13; Fig. 2c, right part) underlining the assumptions of our theoretical model.

Thus, treatment with TMZ significantly reduced total cell numbers of LN229 cells, while the share of dormant cells within the culture, as detected by the dormancy marker H2BK, was drastically elevated. The incidence of conversion to dormancy did not depend on cell densities in our particular experimental setting.

### Treatment schedules

**Fig. 3:**
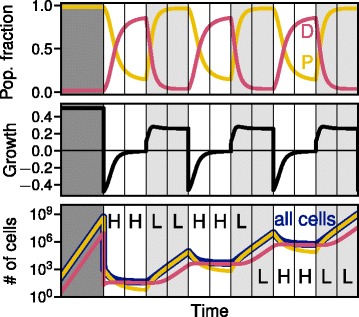
Impact of cyclic treatment on the cell population. In each treatment interval, either high dosage (H) or low dosage (L) treatment is applied.Top: Population composition between dormant (D) and rapidly proliferating (P) cells displayed by the relative fraction of both phenotypes under changing conditions. Middle panel: Average growth rate of the whole population under changing treatment condition. A negative growth rate only occurs in phases of strong treatment. Bottom panel: Absolute number of all tumor cells, P and D phenotype, assuming exponential growth with the given average growth rate (parameters: dormant cell growth rate *ε* = 0, conversion rate *σ* = 0.01, high dosage effect *λ*_*H*_ = 1, low dosage effect *λ*_*L*_ = 0.25)

Next, we use our model to analyze the dynamics of the population composition for periodically changing treatment conditions. One example trajectory for a growth rate for D cells *ε* = 0 and a conversion rate between phenotypes *σ* =0.1 is depicted in Fig. 3. The fraction of D and P cells in the population alternates between the fixed points corresponding to the momentary treatment condition. The trajectory starts with a phase of no treatment, which is characterized by a high average growth rate and a cell population composition of mostly P cells and only very few D cells. After the first phase of unconstrained growth large parts of the tumor are removed (e.g. by surgery), leaving only a small number of cancer cells. Under the following high dosage treatment conditions, the dormant phenotype has the highest fitness and takes over the population. The relative fraction of D cells will increase until the steady state under high treatment conditions is reached. The impact of treatment on P cells leads to a strong initial decline in average growth rate, until the population has a significant proportion of dormant cells and the growth rate starts to recover slightly.

Under the following low dosage treatment conditions, P cells (making up a small fraction of the whole population at the end of the high dosage treatment) are less affected by the treatment and now grow faster. The average growth rate will have a maximum when then relative fraction of P cells in the population is still low, since they have a competitive advantage over D cells, and then declines afterwards towards an equilibrium well above the high dosage growth rate. Accordingly, the total number of cells increases strongly again in this regime.

Switching the order of high dosage and low dosage treatment only has a small effect on total number of cells: If treatment starts with low dosage, the system will go into a state with a slightly higher fraction of dormant cells, which makes it less susceptible to the following high dosage treatment. Starting with low dosage therefore does not help to reduce the tumor size.

**Fig. 4:**
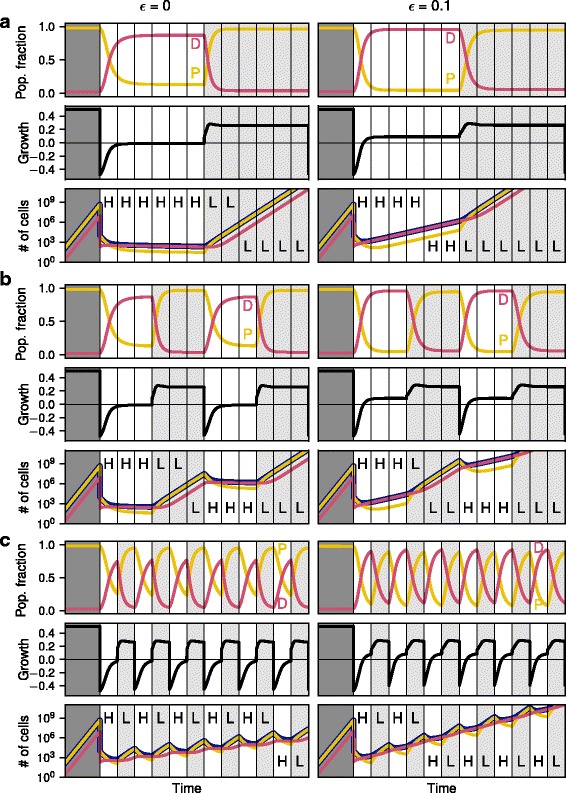
Comparison of the effect of different treatment cycle lengths on population composition, average growth rate and number of cells, similarly to Fig. 3. All panels on the left have a D cell growth rate of *ε* = 0, whereas all panels on the right have *ε* = 0.1. **a** The top row shows the case of high dosage treatment followed by a sustained low dosage treatment. **b** The middle panels use a relatively slow switching between high dosage and low dosage treatment, whereas the bottom panel **c** shows very fast switching. All other parameters as in Fig. 3

In Fig. 4 we compare three different treatment schedules: just one switch from initial high (H) dose to low (L) dose treatment (HHHHHHLLLLLL, Fig. 4a, each instance of the letter H or L corresponds to the same time interval), slow cyclic switching (HHHLLL, Fig. 4b), and fast cyclic switching (HLHL, Fig. 4c) for two different growth rates of D cells (left panels *ε* = 0 and right panels *ε* = 0.1). In case of only switching once, the fixed points for each treatment are quickly reached. At high dosage treatment the number of cells increases very slowly or even decreases. In the following low treatment phase, however, P cells take over growing particularly fast and jeopardizing any positive effect from the previous strong treatment. This is true for both treatment strengths of the high dosage phase.

For the fast switching treatment schedule (HLHL, Fig. 4c), the fixed point of the population proportion is not reached before the treatment changes again. Therefore the population dynamics stays between the two stable fixed points for the two treatment regimes, but does not reach them. By contrast, in the slow treatment switching regime (HHHLLL, Fig. 4b) the fixed points for both high and low dosage treatment are reached such that the composition of the cell population essentially resembles the case of just one switching event (Fig. 4a). However the time spent at these fixed points is still significantly reduced compared to only a single switch.

The bottom panels of Fig. 4a, b and c show the total number of cells based on the average fitness of the population under the assumption of exponential growth. When the growth rate is positive the cell population grows, otherwise it shrinks. Interestingly, the average growth rate of the population is well below zero only for a short period during the high dosage treatment and only if the share of P cells is still very high and the fraction of D cells in the population is small. However, in this regime the fitness recovers fast and approaches equilibrium with an average fitness close to zero, such that the total number of cells does not change anymore. The strongly negative growth rate directly after switching to the high dosage treatment is therefore the reason why the number of cells for quickly changing treatment regimes is significantly smaller than for slowly changing treatment cases.

### Population growth

**Fig. 5:**
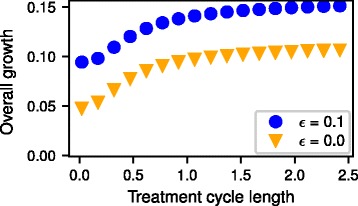
Overall growth rate for different treatment cycle length and for two different growth rates of dormant cells *ε* = 0 and *ε* = 0.1. High and low dosage phases are alternating with the given treatment cycle length for in total 30 cycles. The overall growth rate is then calculated from a linear fit to the log-plot. Other parameters as in Figs. 3 and 4

To systematically examine the effect of switching treatment cycles on the growth rate of the population, we analyze the temporal dynamics of the population size for varying treatment cycle durations and two different growth rates of D cells *ε* (Fig. 5). Unsurprisingly, a lower growth rate of D cells has a diminishing effect on the overall growth of the cells. For increasing treatment cycle length cancer cells have an increasing overall growth, while maintaining the same total high and low dosage treatment durations. The overall growth rate approaches a maximum with increasing treatment cycle length when the dynamics reaches equilibrium in each cycle.

Taken together, our mathematical model allows us to theoretically predict the fitness and proportions of rapidly proliferating and dormant tumor cells under different treatment conditions. Strengthening our theoretical assumptions we could exemplarily show the effect of high and low chemotherapy doses on the cell numbers and the proportion of a dormant cell phenotype in cultured GBM cells in vitro. Simulating different therapy schedules, we observed that fast switching of low and high treatment doses yields a lower total tumor cell number at equal total drug dose in comparison to low switching schedules.

## Discussion

In this study, we established a mathematical model and analyzed the proportions of two different cell phenotypes occurring in GBM, rapidly proliferating and dormant cells. Corroborated by experimental data obtained from in vitro experiments with cultured GBM cells, we observed that treatment strength influences the balance between both phenotypes which in turn influences the growth of the whole tumor population. Sequential switching of treatment strength may thus drastically influence the proportion of dormant and rapidly proliferating cells, especially if switching to the next condition takes place before the population dynamics reaches a steady state.

Dormancy in GBM has been shown by the existence of distinct fraction of temporarily non-proliferative cells in murine models [27], as well as by the identification of clones which were able to generate indolent dormant tumors both in subcutaneous and orthotropic intracranial sites [28]. Additionally, dormancy seems to be characterized by specific features in GBM, such as a non-angiogenic phenotype [24, 28, 29], and is influenced by the (micro-)environment e.g. hypoxia [30] and coagulation [31–33]. However, cellular dormancy in tumors is not only regarded as a state to overcome times of adverse conditions but has also been assigned to DNA repair mechanisms [34]. Interestingly, dormant GBM cells are hallmarked by the upregulation of specific genes like angiomotin, ephrin type-A receptor 5 (EphA5), insulin-like growth factor-binding protein 5 (IGFBP5), and histone cluster 1 (H2BK) [24, 25]. We used the latter as a marker to detect dormant cells in our in vitro experiments and show that the proportion of dormant cells increases with increasing chemotherapy concentrations.

As the fitness in the competition for space and resources depends on the proportions of phenotypically different cell subpopulations, we used evolutionary game theory as a framework for our mathematical model. Previous studies discussed game theoretic interactions with more phenotypes for many different types of cancer, including glioma [35], prostate cancer [36], and multiple myeloma [19, 37] or general tumors [38]. Also, evolutionary game theory is often used in spatially structured populations to answer questions about the effect of environmental constraints on tumor composition and invasiveness of cancer cells [20, 39, 40]. However, including spatial structure in order to increase the realism of the model leads to a large number of additional assumptions and potential pitfalls [41, 42]. Other modeling approaches for dormancy in cancer focus on the interaction between the immune system and the tumor [43–45], the effect of angiogenesis [46], or spatial competition between cells [47]. However, these approaches do not explicitly model the conversion between phenotypes and its consequences under therapy with varying strength.

Thus, we decided to simplify our model on several levels: (i) We do not take spatial structure into account. (ii) We abstract from the interaction with the immune system. (iii) We concentrate on two tumor cell phenotypes – rapidly proliferating and dormant cells – although other cell phenotypes, such as fast migrating cells, cells mimicking vasculature, (cancer) stem cells and invading immune cells (e.g. [48–50]), also contribute to the whole tumor mass. (iv) We focus on the fitness of the respective phenotypes rather than the potentially underlying reasons for phenotypical changes (e.g. genetic or epigenetic changes).

An important aspect of our model is the conversion between the different cell phenotypes. Recent studies suggest that dormant cells may originate from “normal” tumor cells by currently intensively investigated mechanisms (e.g. [51–53]). As a fundamental criterion for tumor dormancy, dormant cells need to be able to reawake and start growing again, so that they then phenotypically resemble the rapidly growing cell phenotype. Thus, we introduced a conversion factor *σ* into our model capturing these phenotypical transformation processes. Whether such conversions occur spontaneously or can be induced specifically or randomly by extrinsic or intrinsic mechanisms is poorly understood. We thus assumed a spontaneous event which can be modeled by a constant rate.

Using our theoretical approach we showed that dependent on the applied treatment strength an equilibrium balance between rapidly proliferating cells and dormant cells is eventually reached. At this fixed point and with low dosage or no treatment, mostly rapidly proliferating cells dominate the population, similar to the findings of Basanta and colleagues [35]. At stronger treatment, the fraction of dormant cells becomes successively larger, yielding a lower growth rate of the whole tumor. However, high dosage treatment cannot be applied for longer time periods, as it causes severe side effects (for GBM: [3]). Hence, we focused on alternative treatment schedules.

Several previous models discuss the effect different treatment schedules on various aspects of the cancer growth like angiogenesis [44, 54] or evolution of resistance [55]. Here, either the dosage and timing or the type of the chemotherapeutic drug is varied, which can have a massive effect on the growth of the tumor. Accordingly, we combine sequential cycles of low and high dose treatment with different durations. Thereby, we observed that the total growth of the tumor is considerably lower for fast switching compared to a slow switching scheme.

## Conclusion

In this study, we have developed a theoretical model to predict the tumor growth kinetics under different treatment strengths including a dormant cell phenotype and underlined our theoretical approach with experimental data. Using our model which allows for phenotypic conversion, we could simulate how different tumor cell phenotypes proportionally contribute to the growing tumor mass in cycling treatment schedules. Additionally, we could observe that switching between high and low dosage treatment (with equal total treatment amounts) remarkably affects tumor growth in a frequency dependent way.

Thus, the dynamic proportions between cell phenotypes should be taken into account in the optimization of treatment schedules in order to control tumor growth.

## Abbreviations

D: Dormant cells
DMSO: Dimethyl sulfoxide
FCS: Fetal calf serum
GBM: Glioblastoma *multiforme*
P: Rapidly proliferating cells
SD: Standard deviation
TMZ: Temozolomide

## Footnotes

1
https://www.scipy.org/
